# Crucial Role of *Legionella pneumophila* TolC in the Inhibition of Cellular Trafficking in the Protistan Host *Paramecium tetraurelia*

**DOI:** 10.3389/fmicb.2018.00800

**Published:** 2018-04-25

**Authors:** Takashi Nishida, Naho Hara, Kenta Watanabe, Takashi Shimizu, Masahiro Fujishima, Masahisa Watarai

**Affiliations:** ^1^The United Graduate School of Veterinary Science, Yamaguchi University, Yamaguchi, Japan; ^2^Department of Sciences, Graduate School of Sciences and Technology for Innovation, Yamaguchi University, Yamaguchi, Japan; ^3^National BioResource Project, Japan Agency for Medical Research and Development, Tokyo, Japan

**Keywords:** *Legionella pneumophila*, *Paramecium*, TolC, cellular trafficking, symbiosis

## Abstract

*Legionella pneumophila* is a facultative intracellular Gram-negative bacterium, which is a major causative agent of Legionnaires’ disease. In the environment, this bacterium survives in free-living protists such as amoebae and *Tetrahymena*. The association of *L. pneumophila* and protists leads to the replication and spread of this bacterium. Thus, from a public health perspective, their association can enhance the risk of *L. pneumophila* infection for humans. *Paramecium* spp. are candidates of natural hosts of *L. pneumophila*, but their detailed relationships remain unclear. In the present study, we used an environmental strain, *L. pneumophila* Ofk308 (Ofk308) and *Paramecium tetraurelia* st110-1a to reveal the relationship between *L. pneumophila* and *Paramecium* spp. Ofk308 was cytotoxic to *P. tetraurelia* in an infection-dependent manner. We focused on TolC, a component of the type I secretion system, which is a virulence factor of *L. pneumophila* toward protists and found that cytotoxicity was dependent on TolC but not on other T1SS components. Further, the number of bacteria in *P. tetraurelia* was not associated with cytotoxicity and TolC was not involved in the mechanism of resistance against the digestion of *P. tetraurelia* in Ofk308. We used a LysoTracker to evaluate the maturation process of *P. tetraurelia* phagosomes containing Ofk308. We found that there was no difference between Ofk308 and the *tolC*-deletion mutant. To assess the phagocytic activity of *P. tetraurelia*, Texas Red-conjugated dextran-uptake assays were performed. Ofk308 inhibited phagosome formation by *P. tetraurelia* through a TolC-dependent mechanism. Further, we evaluated the excretion of *Legionella*-containing vacuoles from *P. tetraurelia*. We found that *P. tetraurelia* failed to excrete undigested Ofk308 and that Ofk308 remained within cells through a TolC-dependent mechanism. Our results suggest that TolC is essential for *L. pneumophila* to remain within *Paramecium* cells and to show cytotoxicity. Because of the high mobility and high cell division rate of *Paramecium* spp., living with *Paramecium* spp. would be beneficial for *L. pneumophila* to expand its habitat. To control Legionaries’ disease, understanding the ecology of *L. pneumophila* in the environment is essential.

## Introduction

*Legionella pneumophila* is a facultative intracellular Gram-negative bacterium that is the major causative agent of Legionnaires’ disease (Cunha et al., 2016), which is a severe pneumonia and a mild flu-like illness called Pontiac fever. The infection of humans occurs by inhalation of a *Legionella*-containing aerosol. Once *L. pneumophila* enters the lung, it invades alveolar macrophages and replicates within *Legionella*-containing vacuoles (LCVs) derived from host phagosomes (Isberg et al., 2009) that serve as a suitable niche for bacterial replication.

*Legionella pneumophila* normally inhabits natural or artificial aquatic environments where it can survive for long periods as free-living forms as well as in biofilms (Fliermans et al., 1981; Paszko-Kolva et al., 1992; Declerck et al., 2007). Further, *L. pneumophila* can survive in free-living protists. Amoebae are protistan hosts of *L. pneumophila* in bodies of fresh water (Rowbotham, 1980), and evidence indicates that *Tetrahymena* serves as its host (Fields et al., 1984). In the environment, the association of *L. pneumophila* and protists leads to the replication and spread of this bacterium as well as to the development of antibiotic resistance (Barker et al., 1995; Winiecka-Krusnell and Linder, 1999). Thus, from a public health perspective, their association can enhance the risk of *L. pneumophila* infection for humans.

Many bacteria other than *L. pneumophila* are resistant to protists (Greub and Raoult, 2004), including human pathogenic intracellular bacteria such as *Coxiella burnetii* and *Listeria monocytogenes* as well as nonpathogenic bacteria. Protistan hosts are thought to represent a primary evolutionary factor for the acquisition and maintenance of virulence toward humans (Al-Quadan et al., 2012). Because of the similarities of the digestive process between macrophages and protists, certain mechanisms to resist protist digestion help bacteria to survive in macrophages. Thus, association between bacteria and protists can cause the emergence of new pathogenic bacteria, because nonpathogenic bacteria may acquire a pathogenic phenotype within protistan hosts. To control current and future pathogenic bacteria as well as *L. pneumophila*, it is extremely important to understand and identify the association between environmental bacteria and protistan hosts.

*Paramecium* spp. are ciliates that exist widely in freshwater environments and are appreciated as model organisms for the analysis of cellular and molecular biology, including phagocytosis and exocytosis (Steinman et al., 1983; Plattner, 2010). Further, *Paramecium* spp. are used to study endosymbiosis, because they possess several endosymbionts (Görtz and Fokin, 2009), and symbionts of *Paramecium* spp. affect the host’s phenotype. For example, *Paramecium caudatum* can acquire resistance to salinity (Duncan et al., 2010) and heat-shock (Hori and Fujishima, 2003; Fujishima et al., 2005) if infected with *Holospora* spp. Previously, we found that *Paramecium* spp. are candidates of a natural host of *L. pneumophila* (Watanabe et al., 2016). *L. pneumophila* have resistance to *Paramecium* digestion and several *L. pneumophila* strains show cytotoxicity toward *Paramecium* spp. *Paramecium* spp. may increase the risk of *L. pneumophila* infection for humans as well as other protistan hosts such as amoebae and *Tetrahymena.* However, it is still unclear whether *L. pneumophila* establishes endosymbiosis with *Paramecium* spp. in the environment.

Type I secretion systems (T1SSs) are widespread in pathogenic Gram-negative bacteria such as *Escherichia coli, Vibrio cholerae*, and *Bordetella pertussis* (Thomas et al., 2014). This secretion machinery comprises three components that transport substrates to the exterior in one step across both lipid bilayers. The first reported protein secreted through T1SS is the hemolysin A (HlyA), which is produced by certain *E. coli* strains (Goebel and Hedgpeth, 1982). HlyA is a member of the repeats-in-toxin (RTX) family that is the most extensively studied. Further, *L. pneumophila* employs T1SS, and RtxA serves as its substrate in *L. pneumophila.* The association of T1SS and RtxA with virulence of *L. pneumophila* such as invasion, intracellular growth, and pore-forming activities toward amoebae has been revealed (Fuche et al., 2015). In addition, TolC, the outer membrane protein of T1SS, plays roles in virulence and multidrug resistance (Ferhat et al., 2009). TolC is a trimeric membrane protein and forms a long channel that protrudes deeply into the periplasm. TolC is composed of a short β-barrel (outer membrane) and a long α-helical (periplasmic) structure (Koronakis et al., 2000). TolC forms the T1SS by interacting with an ATP-binding cassette transporter and a membrane fusion protein that resides in the inner membrane. TolC couples with numerous inner membrane or periplasmic proteins and forms efflux systems such as the AcrAB-TolC efflux pump (Symmons et al., 2015). These efflux systems transport diverse molecules such as virulence-associated proteins, antibiotics, and detergents (Zgurskaya et al., 2011). However, we are unaware of published studies on the role of TolC in the relationship between *L. pneumophila* and *Paramecium* spp.

In the present study, we focused on the role of TolC in the association between *L. pneumophila* and *Paramecium* spp. We found that TolC was essential for *L. pneumophila* to remain within *Paramecium* cells. Our results suggest that *L. pneumophila* employs a TolC-dependent mechanism to survive within *Paramecium* spp. in the environment.

## Materials and Methods

### Bacterial Strains and Culture Conditions

All bacterial strains used in this study are listed in **Table 1**. *L. pneumophila* strains were cultured at 37°C on *N*-(2-acetamido)-2-aminoethanesulfonic acid-buffered charcoal yeast extract agar (BCYE) or in the same medium without agar and charcoal (AYE; Nishida et al., 2017). *E. coli* strains were cultured in Lysogeny Broth (LB; Nacalai Tesuque, Kyoto, Japan) or on LB containing 1.5% agar (Wako, Tokyo, Japan). Media were supplemented with chloramphenicol (10 μg/mL, Wako), kanamycin (30 μg/mL, Wako), and ampicillin (250 μg/mL, Wako) as required.

**Table 1:** Bacterial strains and plasmids used in this study.

Strain	Characteristics	Source or reference
***Legionella pneumophila***		
Ofk308	Isolated from environmental water	Tachibana et al., 2013
Ofk308 Δ*tolC*	*tolC*-deletion mutant of Ofk308	This work
Ofk308 Δ*tolC*/*tolC*	Ofk308 Δ*tolC* carrying pAM239-TolC	This work
***Escherichia coli***		
DH5a	Φ*80lacZ*Δ*M15*, Δ(*lacZYA-argF)U169*, *recA1*, *endA1*, *hsdR17*, *supE44*, *thi-1*, *gyrA96*, *relA1*	Takara
DH5a λ pir	DH5α (λ pir) *tet*::Mu *recA*	Takara
JM109	*recA1*, *endA1*, *gyrA96*, *thi-1*, *hsdR17*, *e14-(mcrA-)*, *supE44*, *relA1*, Δ*(lac-proAB)*	Takara
**Plasmids**		
pAM239-GFP	pMMB-derived vector encoding GFP, CmR	Watarai et al., 2001
pAcGFP	pUC19-derived vector encoding AcGFP1, AmpR	Takara
pSR47s	*ori* R6K *ori* TRP4 *sacB*, KmR	Andrews et al., 1998
pAM239-TolC	pAM239 vector expressing TolC, CmR	This work

### *P. tetraurelia* and Culture Conditions

*Paramecium tetraurelia* st110-1a (ID: PT041001A) was provided by the Symbiosis Laboratory, Yamaguchi University, with support, in part, from the NBRP. Culture and maintenance were previously described (Fujishima et al., 1990). Briefly, the culture medium used was 2.5% (w/v) fresh lettuce juice in Dryl’s solution (Dryl, 1959) inoculated with a nonpathogenic strain of *Klebsiella pneumoniae* the day before use. The cultivation was performed at 25°C. Cells at the stationary phase of growth (20–24 h after the last feeding) were used for the experiments.

### Construction of Deletion Mutants and Complementary Strains

Each deletion mutant was constructed using the homologous-recombination method. Briefly, two PCR fragments were cloned into SalI/NotI or BamHI-cleaved pSR47s (Andrews et al., 1998) using an In-Fusion HD Cloning Kit (Takara, Tokyo, Japan). Fragment 1 was a 1,500 or 2,000 bp fragment spanning a site located upstream of the 5’ end of each target gene. Fragment 2 was a 1,500 or 2,000 bp fragment spanning a site located downstream of the 3’ end of each target gene. These fragments were amplified using PCR. Each plasmid was introduced into *E. coli* DH5a λ pir and subsequently transferred into Ofk308 using electroporation with a Gene Pulser electroporator (Bio-Rad Laboratories, Hercules, CA, United States) in 10% glycerol at 2.5 kV/25 μF. Isolation of in-frame deletion mutants by positive selection for sucrose resistance has been described (Andrews et al., 1998). The *tolC* complementary strain was constructed by cloning a PCR fragment of *tolC* into PstI/EcoRI-cleaved pAM239-GFP (green fluorescence protein) using a DNA Ligation Kit (Takara). This *tolC*-inserted plasmid, pAM239-TolC, was introduced into *E. coli* DH5a and subsequently transferred into *L. pneumophila* using electroporation. GFP or TolC expression in *L. pneumophila* was induced by adding isopropyl-β-D-thiogalactopyranoside (1 mM, Wako) to AYE.

All primers and plasmids used in this work are listed in **Tables 1**, **2**. Plasmid DNA from *E. coli* DH5a was prepared using a QIAGEN Plasmid Mini Kit (QIAGEN, Hilden, Germany). Restriction enzymes (Takara) were used according to the manufacturers’ protocols.

**Table 2:** Primers used in this study.

Primer	Sequence	Target region
tolCuF	ACCGCGGTGGCGGCCGCTGCGAGTGGCAATTGC	Upstream of the 5′ end of *tolC*
toCuR	CGAATTGGATCCλTTTAGGTTTTCTTATGTC	
tolCdF	ACCTλTTTGGATCCTCGAGCTTTCCCTAGλAC	Downstream of the 3′ end of *tolC*
tolCdR	ATCCTCTAGAGTCGCλATGCTCTGGTGTTCC	
lssBDuF	ATCCTCTAGAGTCGACTTACCAGATTGCTGATGC	Upstream of the 5′ end of *lssB*
lssBDuR	TTGCTTAATGATTGTCTTCTCGGAGTATTC	
lssBDdF	ACAATCATTAAGCAATCAGCACTTAGAGAG	Downstream of the 3′ end of *lssD*
lssBDdR	ACCGCGGTGGCGGCCGCGTGATTCCAGCGAATTAG	
rtxAuF	ATCCTCTAGAGTCGACCAAGCGATAAGGTAATAATTG	Upstream of the 5′ end of *rtxA*
rtxAuR	GTTCATCGTTCTGTCCTCλGTTTACTATT	
rtxAdF	GACAGAACGATGAACCCATTACATTGGTG	Downstream of the 3′ end of *rtxA*
rtxAdR	TAGAACTAGTGGATCCGCAGAAGAGCGTATGCCA	
tolCcF	CGAATTGAATTCGTTTTCTAGGGλGCTCG	*tolC*
tolCcR	CGAATTCTGCAGTGTGAATGAATCTTTTCC	

### Cytotoxicity Assay and Determination of the Bacterial Loads in *P. tetraurelia*

Cytotoxicity assays and determination of bacterial loads were performed as previously described (Watanabe et al., 2016). Briefly, Ofk308 or each mutant strain was added to *P. tetraurelia* in 1.5-mL tubes and then incubated at 25°C. After incubation, viable *P. tetraurelia* was counted using microscopy. To determine the bacterial load, *P. tetraurelia* was infected with each strain of *L. pneumophila* at multiplicity of infection [MOI] = 20,000. After incubation at 25°C, *P. tetraurelia* was washed five times with 5 mL of fresh lettuce juice in Dryl’s solution through a 15-μm pore nylon mesh to remove extracellular bacteria. Samples were further treated at 50°C for 30 min to purge the *K. pneumoniae* fed to *P. tetraurelia*. Colony-forming units were determined using serial dilution on BCYE. In infections with *E. coli* JM109, incubation at 50°C was omitted, and LB-containing ampicillin (250 μg/mL) was used.

### Fluorescence Microscopy

GFP-expressing bacteria were added to *P. tetraurelia* at MOI = 20,000, which were then incubated at 25°C for 30 min to 48 h. *P. tetraurelia* was fixed with 4% paraformaldehyde in PBS for 10 min at room temperature. Images of fluorescence were obtained using a FluoView FV100 confocal laser scanning microscope (Olympus, Tokyo, Japan).

When LysoTracker (Life Technologies, Carlsbad, CA, United States) was used, *P. tetraurelia* was fixed 30 min after infection. After fixation, samples were washed twice with PBS and then incubated with LysoTracker (50 nM) for 30 min. LysoTracker-positive LCVs were counted using microscopy, and the data are shown as an average of three fields.

When a Texas Red-conjugated dextran (TRDx, Thermo Fisher Scientific, MA, United States) was used, samples were washed 1 h after infection to remove extracellular bacteria using a nylon mesh as described above. TRDx (50 μg/mL) was added to *P. tetraurelia*. At each sampling time, *P. tetraurelia* was fixed and washed twice with PBS. The number of TRDx-containing vacuoles in individual *P. tetraurelia* was counted and expressed as the average of 30 *P. tetraurelia* cells.

### Observations of Individual *P. tetraurelia* Under NiCl_2_-Induced Paralysis

*Paramecium tetraurelia* was infected with *L. pneumophila* at MOI = 20,000 and then incubated at 25°C for 1 h. After washing as described above, NiCl_2_ (2 mM) was added and the cells were incubated for 10 min at room temperature. After incubation, the *P. tetraurelia* cells were collected and transferred into new media supplemented with 0.2 mM NiCl_2_. Samples were immediately transferred to a 48-well plate. An IX75 inverted fluorescence microscope was used to observe and photograph the cells at 5 min intervals. The number of LCV-containing *P. tetraurelia* in 50 cells was counted at 0 and 40 min.

### Statistical Analyses

Statistical analyses were performed using the Tukey–Kramer test or the Student’s *t*-test. Statistically significant differences between groups were accepted at *P* < 0.05 or *P* < 0.01. Data are presented as the average of three identical experiments, and the error bars shown in the figures represent SDs.

## Results

### *L. pneumophila* Ofk308 Exhibits Cytotoxicity Toward *P. tetraurelia*

*Paramecium* spp. feed on bacteria and bacteria uptaken by *Paramecium* are normally digested. On the other hand, *L. pneumophila* Ofk308 (Ofk308) showed cytotoxicity toward several *Paramecium* strains (Watanabe et al., 2016). However, the bacterial properties in *Paramecium* were not evaluated. Then, we evaluated intracellular localization of Ofk308. As a result of infection assays in which GFP-expressing Ofk308 was added to 93 strains of *Paramecium* spp. (MOI = 10,000), the intracellular localization of Ofk308 was clearly observed in *P. tetraurelia* strains (data not shown). Therefore, we decided to use *P. tetraurelia* st110-1a as a model to analyze the relationship between Ofk308 and *Paramecium* spp. in the present study.

**FIGURE 1:**
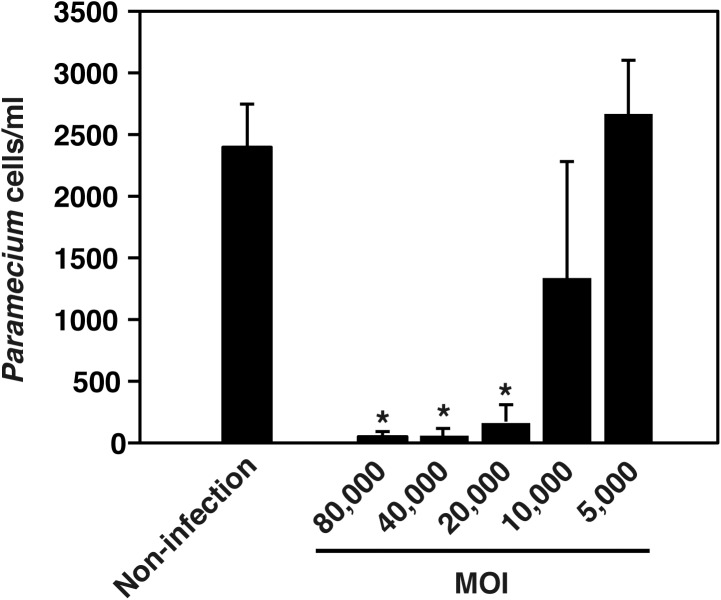
*L. pneumophila* Ofk308 exhibits cytotoxicity toward *P. tetraurelia* in an MOI-dependent manner. The number of *P. tetraurelia* 48 h after infection. *P. tetraurelia* was infected with Ofk308 at MOIs = 5,000, 10,000, 20,000 40,000, and 80,000. Data are expressed as the average of three identical experiments, and the error bars represent SDs. Asterisks indicate statistically significant differences compared with non-infection (^∗^*P* < 0.05).

Ofk308 exhibits cytotoxicity toward several *Paramecium* strains in an MOI-dependent manner (Watanabe et al., 2016). Therefore, the cytotoxicity of Ofk308 toward *P. tetraurelia* was assessed. *P. tetraurelia* was infected with Ofk308 at different MOIs, and viable *P. tetraurelia* was counted 48 h after infection. At MOI ≤ 5,000, the number of viable *P. tetraurelia* was the same as that of uninfected *P. tetraurelia*. However, at MOI = 10,000, the number of viable *P. tetraurelia* tended to decrease, and at MOI ≥ 20,000, the number of viable *P. tetraurelia* decreased significantly compared with uninfected *P. tetraurelia* (**Figure 1**). These results suggest that Ofk308 is cytotoxic to *P. tetraurelia* in an MOI-dependent manner.

### TolC Plays a Crucial Role in the Cytotoxicity of *L. pneumophila* Ofk308

**FIGURE 2:**
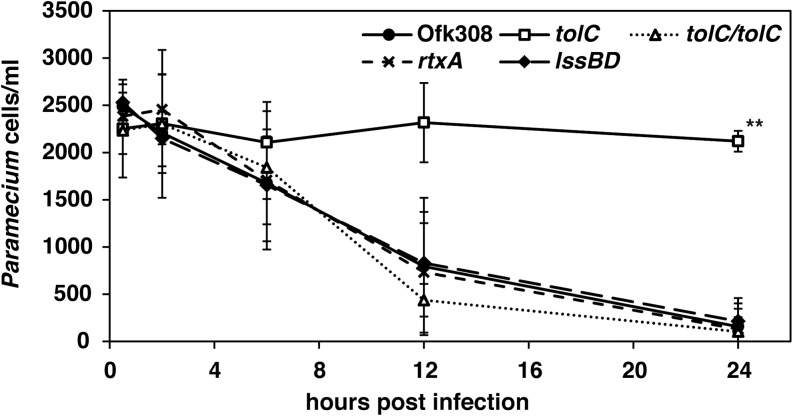
The *tolC*-deletion mutant of *L. pneumophila* Ofk308 is defective for cytotoxicity. *P. tetraurelia* was infected with Ofk308, the *tolC*-deletion mutant (*tolC*), the lssBD-deletion mutant (*lssBD*), the rtxA-deletion mutant (*rtxA*), or the *tolC*-complementary strain (*tolC/tolC*) at MOI = 20,000. Data are presented as the average of three identical experiments, and the error bars represent SDs. Asterisks indicate statistically significant differences compared with Ofk308 (^∗∗^*P* < 0.01).

Type-IV secretion systems of *L. pneumophila* play a major role in its replication within mammalian macrophages and amoebae (Al-Quadan et al., 2012). However, the lack of Type-IV secretion systems does not affect the cytotoxicity of Ofk308 toward *P. caudatum* (Watanabe et al., 2016). Therefore, we focused on T1SS, which is a virulence factor of *L. pneumophila* to amoebae (Fuche et al., 2015). For this purpose, we constructed deletion mutants of *tolC*, *lssBD*, and *rtxA*. TolC is an outer membrane component of T1SS. LssBD serves as the inner membrane and periplasmic components of T1SS, and RtxA is a substrate of T1SS (Fuche et al., 2015). Using these strains, we examined the cytotoxicity toward *P. tetraurelia* at MOI = 20,000. The *tolC*-deletion mutant lost its cytotoxicity toward *P. tetraurelia*, and its complementary strain recovered cytotoxicity comparable to that of the parental strain Ofk308 (**Figure 2**). However, the number of viable *P. tetraurelia* decreased to the same degree as Ofk308 when the *lssBD*- and *rtxA*-deletion mutants were infected. These results indicate that the cytotoxicity of Ofk308 is dependent on TolC but not on T1SS.

### The Cytotoxicity of *L. pneumophila* Ofk308 Is Independent on the Number of Bacteria Within *P. tetraurelia*

**FIGURE 3:**
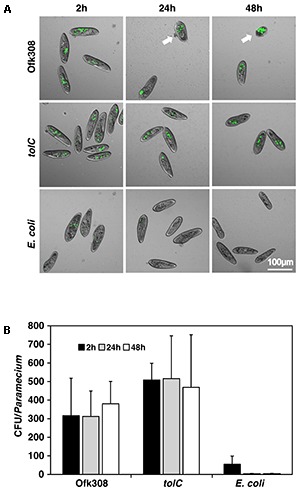
The number of *L. pneumophila* in *P. tetraurelia* is constant. **(A)** GFP-expressing bacteria, Ofk308, the *tolC*-deletion mutant (*tolC*), and *E. coli* JM109 in *P. tetraurelia* 2, 24, and 48 h after infection at MOI = 20,000. Arrows point to unnaturally shaped *P. tetraurelia*. **(B)** Number of bacteria per *P. tetraurelia*. Data are expressed as the average of three identical experiments, and the error bars represent SDs.

To investigate how Ofk308 exhibits cytotoxicity toward *P. tetraurelia* through a TolC dependent mechanism, we compared bacterial numbers in *P. tetraurelia* using Ofk308, the *tolC*-deletion mutant, and *E. coli* as a control. From 2 to 48 h after infection at MOI = 20,000, the numbers of *E. coli* decreased but the numbers of Ofk308 and those of the *tolC*-deletion mutant were unchanged (**Figure 3**). Comparable results were observed at lower MOIs (data not shown). In cells infected with Ofk308, the shape of *P. tetraurelia* changed unnaturally, although damage was not observed in cells infected with the *tolC*-deletion mutant. These results suggest that the number of bacteria in *P. tetraurelia* is not associated with cytotoxicity and that TolC is not involved in the mechanism of resistance against the digestion of *P. tetraurelia* in Ofk308.

### *L. pneumophila* Ofk308 Inhibits the Phagocytic Activity of *P. tetraurelia* Through a TolC-Dependent Mechanism

**FIGURE 4:**
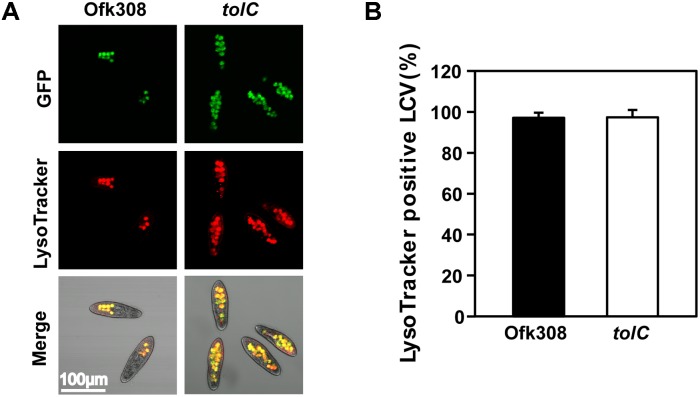
The maturation of host LCVs containing *L. pneumophila* Ofk308 is not inhibited. **(A)** LCV maturation 30 min after infection was evaluated using LysoTracker. Ofk308 or the *tolC*-deletion mutant (*tolC*) was added to *P. tetraurelia* at MOI = 20,000. **(B)** The percentage of LysoTracker-positive LCVs. Data are expressed as the average of three identical experiments, and the error bars represent SDs.

Next, we focused on phagocytic activity of *P. tetraurelia* after mixing with Ofk308 because we hypothesized that the inhibition of phagocytic activity could cause cytotoxicity. *P. tetraurelia* exhibits high phagocytic activity (Plattner and Kissmehl, 2003), and *L. pneumophila* modulates host phagosomes to survive within them (Isberg et al., 2009). In infection of *P. caudatum*, Ofk308 inhibits phagosome–lysosome fusion (PL-fusion; Watanabe et al., 2016). Therefore, we used a LysoTracker to evaluate the maturation of *P. tetraurelia* phagosomes containing Ofk308. We found that there was no difference between Ofk308 and the *tolC*-deletion mutant; 97.1% of LCVs were LysoTracker positive in cells infected with Ofk308 and 97.4% of LCVs were LysoTracker positive in cells infected with the *tolC*-deletion mutant (**Figure 4**).

**FIGURE 5:**
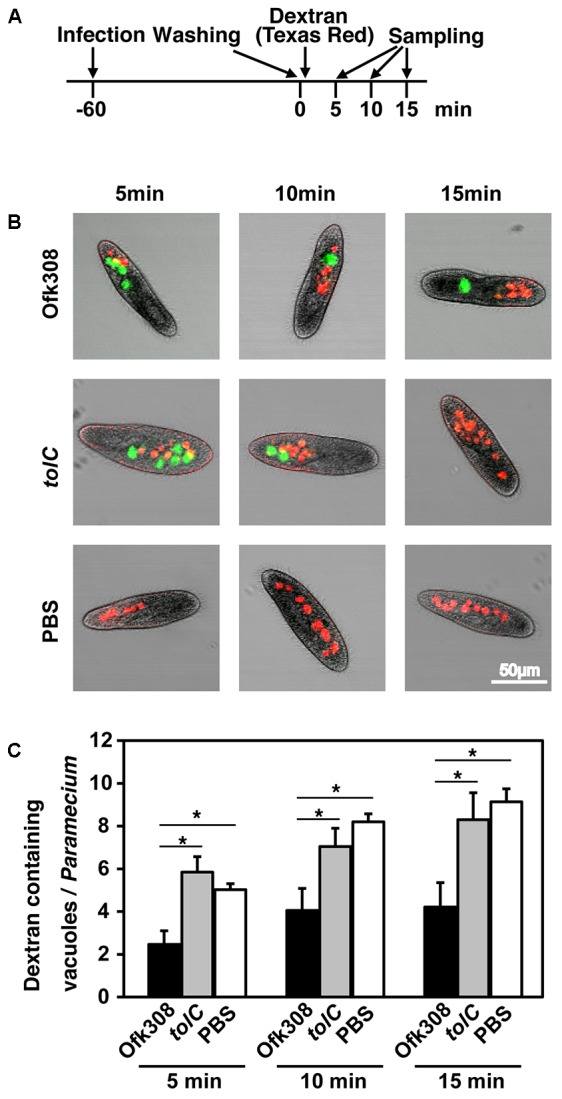
*L. pneumophila* Ofk308 inhibits phagosome formation by *P. tetraurelia* through a TolC-dependent mechanism. **(A)** Bacteria and TRDx were added to *P. tetraurelia* according to this schedule. The number of DCVs was counted at each indicated time point. **(B)** DCVs in *P. tetraurelia* infected with Ofk308 and *tolC* at each indicated time point. DCVs are red, and *Legionella*-containing vacuoles are green. **(C)** The number of DCVs in individual *P. tetraurelia*. Data are expressed as the average of three identical experiments, and error bars represent SDs. Asterisks indicate statistically significant differences (^∗^*P* < 0.05).

Next, to assess the formation of phagosomes, TRDx-uptake assay was performed (according to the schedule shown in **Figure 5A**). Dextran-containing vacuoles (DCVs) represent vacuoles that formed after adding TRDx. In cells infected with Ofk308, 5, 10, and 15 min after adding dextran, the average numbers of DCVs per cell were 2.5, 4.1, and 4.2, respectively. In contrast, in cells infected with the *tolC*-deletion mutant, the average numbers of DCVs were 5.8, 7.0, and 8.3, respectively (**Figures 5B,C**). There were significant differences between Ofk308 and the *tolC*-deletion mutant. These results suggest that Ofk308 inhibits phagosome formation by *P. tetraurelia* through a TolC-dependent mechanism.

### *L. pneumophila* Ofk308 Remains Within *P. tetraurelia* Through a TolC-Dependent Mechanism

**FIGURE 6:**
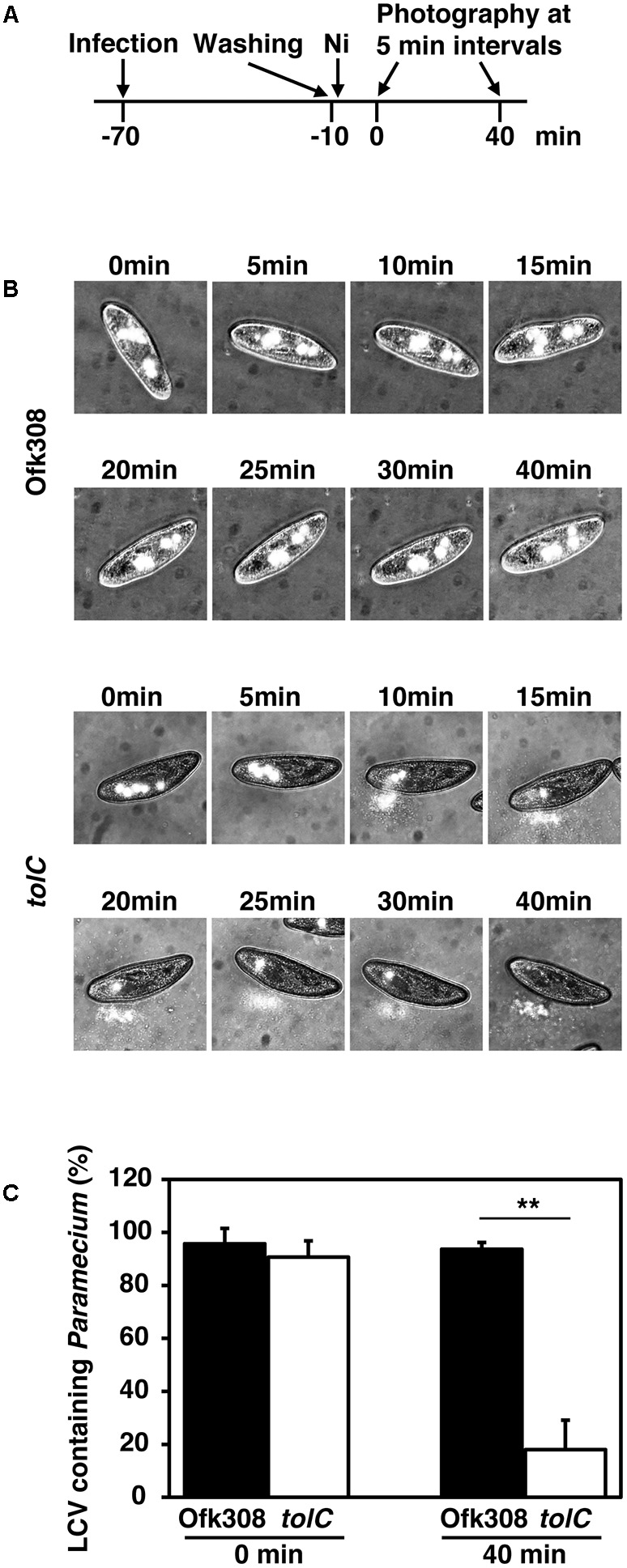
*L. pneumophila* Ofk308 inhibits excretion of LCVs from *P. tetraurelia* through a TolC-dependent mechanism. **(A)** Images of individual *P. tetraurelia* under NiCl_2_-induced paralysis were taken according to this schedule. **(B)** LCVs in individual *P. tetraurelia* at each indicated time point. LCVs are white. **(C)** The percentages of LCV-containing *P. tetraurelia* at 0 and 40 min. Data are expressed as the average of three identical experiments, and the error bars represent SDs. Asterisks indicate statistically significant differences (^∗∗^*P* < 0.01).

Surviving in free-living protists is beneficial for *L. pneumophila*. *L. pneumophila* must inhibit the exocytosis of *Paramecium* to remain within them, because *Paramecium* exhibits high exocytic activity (Plattner and Kissmehl, 2003). We assumed that Ofk308 terminates the exocytic activities of *P. tetraurelia.* However, because of high mobility of *P. tetraurelia*, evaluation of the exocytic activities in individual *P. tetraurelia* is difficult. Therefore, the digestion vacuolar cycles of individual *P. tetraurelia* was observed under NiCl_2_-induced paralysis (according to the schedule shown in **Figure 6A**). By treated with NiCl_2_, *P. tetraurelia* stopped swimming but cytoplasmic streaming was observed; 93.6% of *P. tetraurelia* possessed Ofk308-containing vacuoles for at least 40 min (**Figures 6B,C**). In contrast, the *tolC*-deletion mutant-containing vacuoles were gradually excreted. Finally, most *tolC*-deletion mutants were excreted until 40 min and only 18.0% of *P. tetraurelia* possessed LCVs. These results suggest that *P. tetraurelia* failed to excrete undigested Ofk308 and that Ofk308 remained within cells through a TolC-dependent mechanism.

## Discussion

In the environment, *L. pneumophila* replicates within a protistan host. Previous reports reveal the mechanisms of infection and replication in protists such as amoeba and *Tetrahymena* (Al-Khodor et al., 2009; Richards et al., 2013). However, in these reports, the temperatures used for most infection procedures ranged from 30 to 37°C, because these temperatures are appropriate for the culture of *L. pneumophila* and amoebae. Thus, it is possible that there are other mechanisms, which function at lower temperatures. In the present study, we used *P. tetraurelia* as a model protistan host of *L. pneumophila*. *Paramecium* spp. are candidates of natural host of *L. pneumophila* (Watanabe et al., 2016). *Paramecium* spp. are widely present in fresh water environments but their resistance to high temperature is lower compared with amoeba or *Tetrahymena* (Thatcher and Gorovsky, 1993; Hori and Fujishima, 2003). Thus, all infection assays in our present work were performed at 25°C, which is typical for *Paramecium* spp. culture conditions. Although the cytotoxicity of *L. pneumophila* toward amoeba decreases at lower temperature (Ohno et al., 2008), cytotoxicity of Ofk308 toward *P. tetraurelia* was clearly observed at 25°C in an MOI-dependent manner (**Figure 1**). The association of *L. pneumophila* and *Paramecium* spp. can lead to the replication and spread of *L. pneumophila* in natural aquatic environments. For this reason, our assay likely reflects the natural environmental conditions that are conducive to the survival of *L. pneumophila* and can be helpful to reveal the ecology of *L. pneumophila* in the environment.

The Dot/Icm system of *L. pneumophila* contributes to intracellular survival and replication in amoeba (Richards et al., 2013). However, in our previous report, the Dot/Icm system had no effect on cytotoxicity toward *P. caudatum* (Watanabe et al., 2016). Therefore, we focused on another secretion system, T1SS. Many Gram-negative pathogenic bacteria such as *E. coli* and *B. pertussis* employ T1SS, whose association with bacterial virulence is established (Goebel and Hedgpeth, 1982; Glaser et al., 1988). The role of T1SS in intracellular bacteria has been investigated as well. Several T1SS substrates are present in *Orientia tsutsugamushi* and *Ehrlichia chaffeensis* (Wakeel et al., 2011; VieBrock et al., 2015). T1SS in *L. pneumophila* was investigated, and the roles of T1SS in pore-forming activity and intracellular replication are established (Fuche et al., 2015). In the present study, we evaluated the effects of T1SS on cytotoxicity toward *P. tetraurelia* using a deletion-mutant of T1SS. As a result, in infection with the *tolC*-deletion mutant, decreased cytotoxicity was observed (**Figure 2**). However, other components of T1SS, including the T1SS substrate RtxA, did not affect cytotoxicity. These results suggest that TolC of *L. pneumophila* possesses another function separate from T1SS and that this function may be important in cytotoxicity of *L. pneumophila* toward *P. tetraurelia*. TolC forms several multidrug efflux pumps (Symmons et al., 2015), and TolC of *L. pneumophila* contributes to multidrug resistance (Ferhat et al., 2009). Multidrug efflux pumps are associated with the virulence of *Salmonella enterica* serovar Typhimurium (Buckley et al., 2006; Nishino et al., 2006). Therefore, an efflux pump composed of TolC may provide an important function in cytotoxicity of *L. pneumophila* toward *P. tetraurelia*. Further, *Rickettsia typhi* may employ another TolC-dependent secretion mechanism (Kaur et al., 2012). In this model, an ankyrin repeat-containing protein translocates to the periplasm via the Sec translocon. This protein is secreted via TolC. How TolC recognizes this protein in the periplasm is unknown, and it is unclear if *L. pneumophila* employs a similar secretion mechanism. Further study is therefore required to determine the function of TolC in *L. pneumophila*.

*Legionella*-endosymbiosis modulating factor A (LefA) regulates the relationship between *L. pneumophila* and *Paramecium* spp. (Watanabe et al., 2016). LefA is associated with intracellular replication and inhibition of PL-fusions in *P. caudatum*. In mammalian macrophages or amoebae, *L. pneumophila* modifies host phagosomes and avoids PL-fusions (Richards et al., 2013; Xu and Luo, 2013). Therefore, we examined the effects of TolC on intracellular replication and PL-fusions of *P. tetraurelia* with Ofk308. As a result, the same levels of intracellular localization and PL-fusions were observed in infections with Ofk308 and the *tolC*-deletion mutant (**Figures 3**, **4**). These results indicate that Ofk308 avoids *P. tetraurelia* digestion independently of TolC. The mechanism of the resistance to digestion is unclear, but temperature may serve as a key factor, because all experiments were performed at 25°C.

*Paramecium* spp. possess high phagocytic and exocytic activities (Plattner and Kissmehl, 2003). In contrast, *Chlorella*, which is known as an endosymbiont of *P. bursaria*, remains for at least 72 h (Kodama and Fujishima, 2005). The symbiotic relationship between *P. bursaria* and *Chlorella* was well investigated. Then, endosymbionts of *Paramecium* may inhibit phagocytic and exocytic activities. We show here that TolC played a role in the inhibition of the excretion of LCVs (**Figure 6**). This inhibition follows that *L. pneumophila* remains within the host *Paramecium*. Therefore, TolC may represent a crucial factor required for Ofk308 to remain within *P. tetraurelia* and to establish symbiosis.

High concentrations of Ofk308 exhibited cytotoxicity toward *P. tetraurelia* through a TolC-dependent mechanism (**Figures 1**, **2**). In infections with *P. caudatum*, significant intracellular replication of Ofk308 occurs and causes the death of the host *P. caudatum* (Watanabe et al., 2016). Thus, intracellular replication can represent a major cause of death. However, in the present study, such intracellular replication of Ofk308 was not observed in *P. tetraurelia* (**Figure 2**). Therefore, we reasoned that the inhibition of phagosome formation can cause the death of *P. tetraurelia*. After infection with *P. tetraurelia*, Ofk308 inhibited new phagosome formation through a TolC-dependent mechanism (**Figure 5**). In *Paramecium* spp., the membrane used to form new phagosomes is provided by recruitment of cytoplasmic discoidal vesicles that originate, in part, at the cytoproct (Steinman et al., 1983; Guerrier et al., 2017). Thus, excess Ofk308 remaining within *P. tetraurelia* cells can stop the recycling of the phagosome membrane to form new phagosomes. As a result, *P. tetraurelia* may starve and subsequently die because of poor nutrition. Further, the inhibition of phagosome formation can result in inhibition of invasion of other microbes to *Paramecium*. In natural condition, *L. pneumophila* may inhibit phagocytic activities by TolC-dependent mechanism to occupy host *Paramecium*.

## Conclusion

We show here that TolC is essential for *L. pneumophila* to remain within *Paramecium* cells and to show cytotoxicity. The association between *L. pneumophila* and *Paramecium* spp. in the environment can enhance the risk of infection by *L. pneumophila*. Because of the high mobility and high cell division rate of *Paramecium* spp., remaining within *Paramecium* spp. would be beneficial for *L. pneumophila* to expand its habitat. To control Legionaries’ disease, understanding the ecology of *L. pneumophila* in the environment is essential. Our work therefore promises to facilitate further studies focused on the ecology of *L. pneumophila* in the environment.

## Author Contributions

TN and MW conceived and designed the research. TN, NH, KW, and TS performed the experiments and analyzed the data. KW and MF offered advice and technical assistance for carrying out the studies on protists. TN, KW, and MW wrote the paper.

## Conflict of Interest Statement

The authors declare that the research was conducted in the absence of any commercial or financial relationships that could be construed as a potential conflict of interest.
